# Identification of A Novel Antibacterial Peptide from Atlantic Mackerel belonging to the GAPDH-Related Antimicrobial Family and Its In Vitro Digestibility

**DOI:** 10.3390/md17070413

**Published:** 2019-07-12

**Authors:** Clément Offret, Ismaïl Fliss, Laurent Bazinet, André Marette, Lucie Beaulieu

**Affiliations:** 1 Institute of Nutrition and Functional Foods (INAF), Université Laval, Québec, QC G1V 0A6, Canada; 2 Department of Food Science, Faculty of Agricultural and Food Sciences, Université Laval, Québec, QC G1V 0A6, Canada; 3 Department of Medicine, Faculty of Medicine, Cardiology Axis of the Québec Heart and Lung Institute (Hôpital Laval), Laval University Hospital Research Center, Québec, QC G1V 4G2, Canada

**Keywords:** Atlantic mackerel, hydrolysate, antimicrobial peptide, in vitro digestibility

## Abstract

The Atlantic mackerel, *Scomber scombrus*, is one of the most fished species in the world, but it is still largely used for low-value products, such as bait; mainly for crustacean fishery. This resource could be transformed into products of high value and may offer new opportunities for the discovery of bioactive molecules. Mackerel hydrolysate was investigated to discover antibacterial peptides with biotechnological potential. The proteolytic process generated a hydrolysate composed of 96% proteinaceous compounds with molecular weight lower than 7 kDa. From the whole hydrolysate, antibacterial activity was detected against both Gram-negative and Gram-positive bacteria. After solid phase extraction, purification of the active fraction led to the identification of 4 peptide sequences by mass spectrometry. The peptide sequence N-KVEIVAINDPFIDL-C, called Atlantic Mackerel GAPDH-related peptide (AMGAP), was selected for chemical synthesis to confirm the antibacterial activity and to evaluate its stability through *in vitro* digestibility. Minimal inhibitory concentrations of AMGAP revealed that *Listeria* strains were the most sensitive, suggesting potential as food-preservative to prevent bacterial growth. In addition, in vitro digestibility experiments found rapid (after 20 min) and early digestibility (stomach). This study highlights the biotechnological potential of mackerel hydrolysate due to the presence of the antibacterial AMGAP peptide.

## 1. Introduction

In Canada, as in the rest of the world, the Atlantic mackerel, *Scomber scombrus*, is one of the most fished species, with almost 1.5 million tons caught in 2015 [1]. Mackerel fishing pressure doubled between 2009 and 2015, mainly due to the wide distribution, from eastern to western North Atlantic, and the abundance of this pelagic fish. If mackerel fishing is appreciable, by contrast, the utility of this fish remains largely untapped, as it is mainly used as a source of food for human consumption or as bait for crustacean fishery [2]. Moreover, during processing, 30% to 50% of the total mackerel biomass is discarded and remains unexploited. These kinds of low-value marine products, as unused marine food biomass, must be revalued, with more attention given to their biopreservative potential. 

Indeed, many marine organisms, such as fish, constitute a promising source of compounds with high value-added applications. The fish family *Scombridae* is an excellent source of omega-3 fatty acids, such as eicosapentaenoic (EPA) and docosahexaenoic acids (DHA), widely used as additives in infant formula and other food products [3]. These fish species are also known to be good sources of proteins with well-balanced fat and amino acid composition [4]. By using commercial enzymes such as alcalase, neutrase, protease N and Protamex^®^, the conversion of fish into hydrolysate provides access to more valuable proteins and peptides. As reviewed by [1], marine fish subject to chemical or enzymatic hydrolysis provide a rich source of bioactive compounds, such as proteins and peptides. Many of these proteinaceous compounds exhibit biological activities, such as antioxidant [5], antihypertensive [6], and antibacterial activities [7].

Furthermore, in the context of increasing antibacterial resistance, with the emergence of the superbugs coupled to a decrease in new exploitable drugs, the search for antimicrobial peptides (AMPs) from natural sources emerged as a promising alternative in the post-antibiotic era [8,9]. Compared to common antibiotics, AMPs do not trigger the same resistance mechanisms as classical antibiotics and possess a narrow spectrum, strong activity, thermal stability, multiple modes of action, and are non-toxic and generally recognized as safe (GRAS). Native AMPs could play a key role in the innate immune system of both vertebrates and invertebrates, as Glyceraldehyde-3-phosphate dehydrogenase (GAPDH)-related antibacterial peptide [10] and hemoglobin ß chain-related antibacterial peptide [11] have both been identified in scombrids. AMPs produced by protein hydrolysis usually have a molecular weight below 10 kDa, are composed of fewer than 50 amino acids, and have approximately 50% hydrophobic residues [12]. Alcalase hydrolysis of gelatin extracted from tuna skin produced peptide fractions that exhibited antibacterial activity [13]. AMPs have also been identified from Atlantic mackerel after by-product hydrolysis. After purification of hydrolysate generated by the commercial enzyme mixture Protamex^®^, four peptide sequences were identified, exhibiting activity against both Gram positive and negative bacteria [14]. Among these peptides, collagencin, identified from the mackerel collagen hydrolysate, was particularly active against *Staphylococcus aureus* with 67% growth inhibition at 235 µM [15]. Elucidation of the peptide structure allowed the mechanism of action to be identified, indicating that the peptide acted as a carpet on bacterial membranes [15]. Therefore, knowing the characteristics of AMPs generates important interest for biomedical, pharmaceutical and also industrial applications as bio-preservatives. However, although some previous studies may have focused on characterizing peptides from Atlantic mackerel hydrolysate, none evaluated their stability under gastrointestinal tract conditions. 

The aim of this study was to identify and valorize new antimicrobial peptides from mackerel. The main objectives were to (i) detect any antibacterial activity from fractionated hydrolysate on microbial strains of interest in the food and health sectors, (ii) select the active fractions to develop purification methods targeting antimicrobial peptides, (iii) purify peptides by solid-phase extraction and liquid chromatography, (iv) elucidate the amino acid sequences of these peptides and identify them using known databases, (v) validate the resulting peptides by antibacterial assay and minimal inhibitory concentration (MIC) values against bacteria known as industrial spoilers or isolated from human flora, and (vi) investigate the stability of peptides in the upper portion of the human gastrointestinal (GI) tract.

## 2. Results

### 2.1. Chemical Characterization of the Generated Hydrolysate

The composition of mackerel hydrolysate produced by enzymatic hydrolysis of Atlantic mackerel fished in 2016 was characterized. Chemical analysis of the spray-dried hydrolysate showed that over 90% of the hydrolysate was composed of protein (90.35 g ± 0.16). The second most abundant component was ash, at approximately 6% (6.36 g ± 0.01). Moisture was the smallest component, since it was less than 1% (0.90 g ± 0.06). Lipids, which were separated and removed by centrifugation from peptides in the liquid fraction, were not found in the dried hydrolysate.

The composition of amino acids in hydrolysates is presented in order of abundance (g/100 g of hydrolysate) in Table 1. Acidic amino acids, such as glutamic and aspartic acids, were the most abundant, while taurine, cystine and tryptophan were found at low levels of less than 1 g per 100 g of mackerel hydrolysate. 

**Figure 1:**
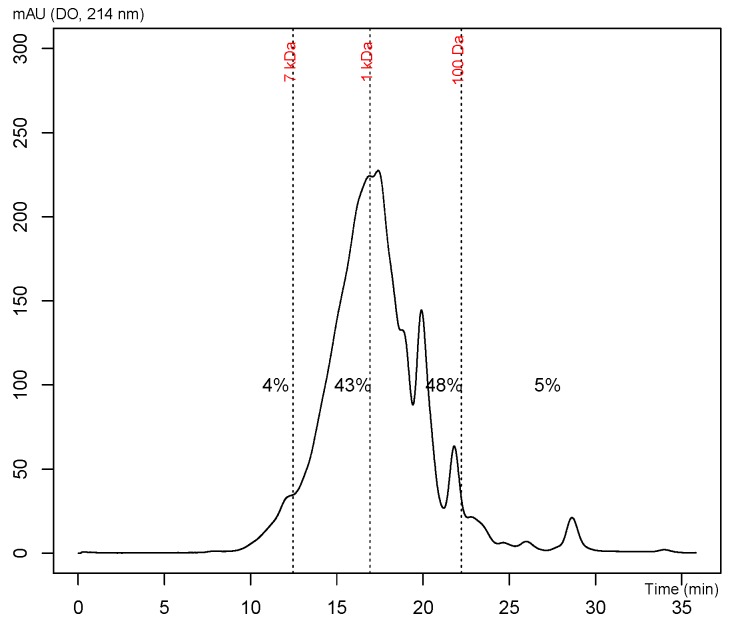
Molecular weight distribution obtained after injection of the mackerel hydrolysate (10,000–200 Da retentate) into the FPLC system.

The molecular weight distribution of peptides was performed by fast protein liquid chromatography system (FPLC) using a Gel Permeation Chromatography (GPC) column on the whole hydrolysate (Figure 1). The proteolytic processing was confirmed by the low percentage (4%) of peptides with mass higher than 7 kDa. The hydrolysate was mainly composed of peptides with a mass of around 1 kDa.

### 2.2. Identification of Antibacterial Peptides

#### 2.2.1. Screening for Antibacterial Activity of Whole Mackerel Hydrolysate and Solid Phase Extraction (Spe) Fractions

**Figure 2:**
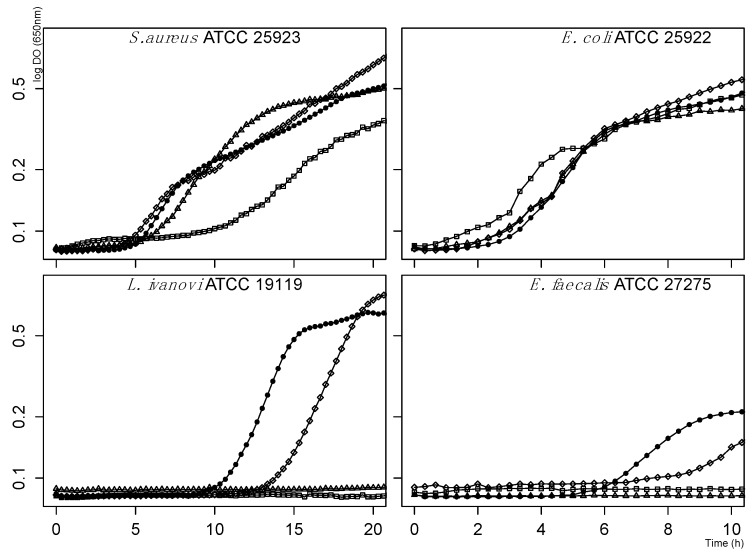
Growth kinetics of bacterial targets in presence of the SPE-C18 fraction at 0 (filled circles), 43 (diamonds), 86 (triangles) and 172 (square) µg/mL.

Mackerel hydrolysates were screened for antibacterial activity against microbial strains of interest in the environmental, food and health sectors. Activity was evaluated by the agar well diffusion assay and the critical-dilution micromethod from whole and fractionated mackerel hydrolysate. At saturation (0.25 g/mL), whole hydrolysate showed no activity by agar well diffusion assay, while the critical-dilution micromethod detected a delay in *Listeria ivanovii* growth. However, after fractionation of hydrolysate, inhibition zones in the agar diffusion assay were observed for the Gram-positive bacteria *Enterococcus faecalis*, *L. ivanovii*, *Staphylococcus aureus* and the fungus *Paecilomyces* spp (data not shown). Optimized SPE fractionation showed that bioactive compounds were eluted with 70% (*v*/*v*) acetonitrile (ACN). The total protein concentration of the active fraction was 1.48 mg/mL. The *L. ivanovii* strain was the most sensitive, since the SPE-C18 fraction completely inhibited growth, reducing it to 86 µg/mL (Figure 2). Consequently, this strain was used to follow the active compounds during the purification process.

#### 2.2.2. Purification Process

**Figure 3:**
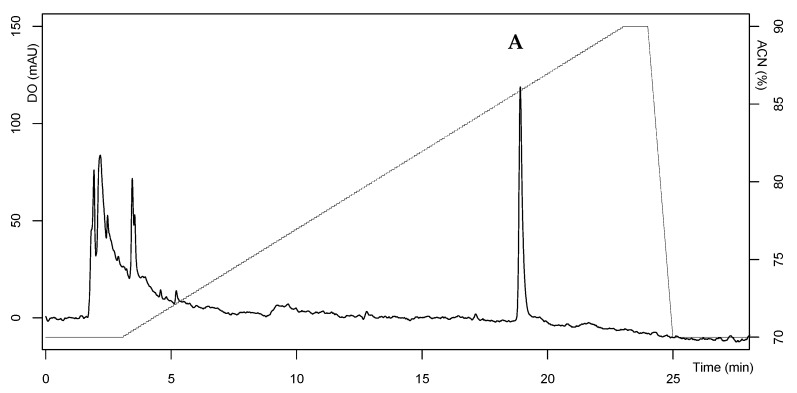
RP-HPLC chromatogram of antibacterial peptide fraction from the second RP-HPLC eluate. The absorbance was monitored at 214 nm and expressed as mAU (arbitrary units of absorbance).

The active fraction from the whole mackerel hydrolysate separated on SPE-C18 cartridges and eluted with 70% ACN was purified by two steps of Reverse-Phase–High-Performance Liquid Chromatography (RP-HPLC). The first step eluted a strong hydrophobic fraction, collected between 15 and 20 min, and the second produced a purified fraction. The active fraction was then tested for antibacterial activity against the most sensitive strain, *L. ivanovii* ATCC 19119. As shown in Table 2, specific activity increased considerably after the first purification step by SPE-C18, restoring 64% of the solubilized hydrolysate activity. However, the SPE-C18 step allowed us to increase specific activity 5405-fold, and total protein (1.48 mg) decreased significantly compared to the whole mackerel hydrolysate solution (1.25 × 10^3^ mg). The first elution yield obtained after the RP-HPLC step was drastically reduced (2%), although activity against the target strain was higher, along with a decrease in protein concentration, resulting in a higher specific activity (14,706). Second purification of antibacterial peptides by RP-HPLC recovered a distinct peak, peak A, eluted by 86% ACN and corresponding to a retention time of 19 min (Figure 3), suggesting strong hydrophobic compounds with high affinity for the reversed phase column. The second RP-HPLC step increased the specific activity of antibacterial peptides (peak A collected) 20,833-fold compared to the initial hydrolysate activity and the amount recovered was 1% of that in the solubilized hydrolysate. Peak A was analyzed by liquid chromatography tandem mass spectrometry (LC-MS/MS) to determine the mass and amino acid sequence.

#### 2.2.3. Characterization of Antibacterial Peptides

The composition of the collected peak was determined by LC-MS/MS, and the results were compared to the Mascot TAX_Percomorphaceae_CI_1489872_20170406 database. With a protein-threshold probability greater than 95%, 46 peptide sequences were identified with lengths between 8 and 18 amino-acid residues. These sequences correlated with a total of 12 different precursor proteins. The search for AMPs in Collection of Anti-Microbial Peptides (CAMP) and Antimicrobial Peptides Database (APD) databases allowed us to distinguish 8 peptide sequences, found only in the CAMP database, with over 75% homology with known AMPs. In silico analyses using Expasy tools showed that 4 of these sequences (Table 3 were predicted to have charge, hydrophobicity and stability index properties like the purified-fraction properties and so might exhibit in vitro activity. The Gravy index, calculated from the hydrophobicity of the amino acids making up the peptides, was particularly significant (>3) for 3 sequences: LILLILLLLKLLLLLI (2), LLILLLLKLLLLLI (3) and LLILLLLLLILLLILLPF (4) because of their leucine and isoleucine content. Peptides (2) and (3) were positively charged due to their lysine amino-acid residue while peptide (4) was neutral. While the identified protein precursors of these three peptides were different, they shared homology (75%) with similar Beta-defensins (CAMPSQ4886-4887). In contrast, the sequence of peptide (1) KVEIVAINDPFIDL was very different from the others and was highly homologous (89%) to the AMP designated as the Yellow fin tuna GAPDH-related antibacterial peptide (YFGAP, CAMPSQ3690). The glutamic and aspartic acids conferred a global negative charge even though the sequence contained a lysine residue. The Gravy index (0.771) was lower than for the three other peptides, indicating weak hydrophobicity. Based on stability, solubility properties, high homology and organism sources, peptide (1) was selected for chemical synthesis to confirm its antibacterial activities. In addition, peptides (2), (3) and (4) could not be synthetized, due to their high leucine content and low solubility.

### 2.3. MIC and Gastrointestinal Digestibility of the Synthetic GAPDH-Related Derived Peptide (1: AMGAP)

#### 2.3.1. MIC

The antibacterial activity of the synthetic Atlantic Mackerel GAPDH-related derived Peptide (AMGAP) was evaluated against pathogenic and non-pathogenic bacteria originating from human commensal flora (Table 4). The MIC was determined for all strains, except for *L. salivarius*, and revealed that the synthetic peptide exhibited a broad spectrum of antibacterial activity, since all the target strains were sensitive—anaerobic Gram-positive strains as well as aerobic Gram-negative strains. The MIC values fell between 1.050 and 0.131 mM, and both *Listeria* strains, *L. ivanovii* and *L. monocytogenes* were the most sensitive. 

#### 2.3.2. Digestibility of the Synthetic GAPDH-Related Derived Peptide (AMGAP) under Dynamic Gastrointestinal Conditions

**Figure 4:**
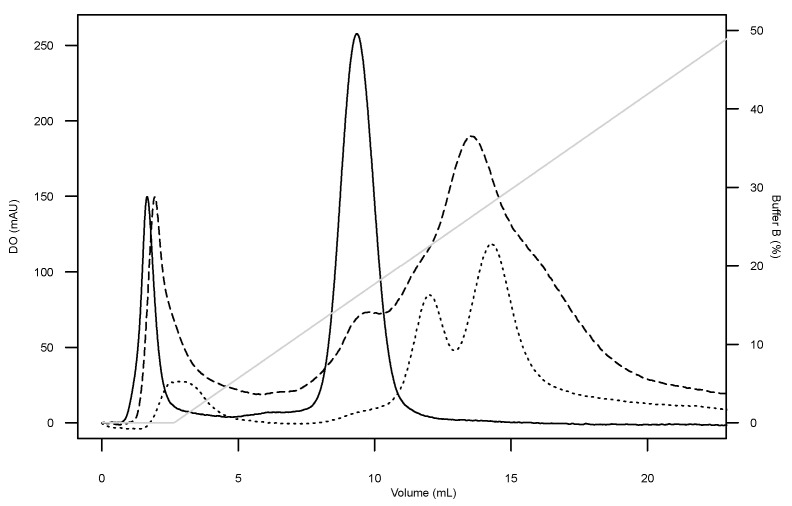
FPLC profile of the synthetic peptide after digestion using dynamic GI TIM-1. Samples collected from the stomach before digestion (solid line) and after 20 min of digestion (short dash), and from the duodenum (long dash).

The digestibility of the synthetic peptide was evaluated for its stability through in vitro dynamic gastrointestinal conditions. After 0, 20, 40, 60 and 120 min of digestion, samples from stomach, duodenum and ileal-delivered effluent were analyzed by FPLC to evaluate the impact of the gastrointestinal process on structural stability of the synthetic peptide (Figure 4). After 20 min of digestion, the intact synthetic peptide could not be detected in either duodenum or ileal-delivered effluent fractions. However, digestibility of the synthetic peptide was observed in the stomach fraction through the appearance of 2 distinct peaks (12 and 14.2 mL of buffer), while before the digestive process, synthetic peptide was eluted at 9.3 mL as a unique peak. The MS analyses confirmed these results detecting only a small proportion (2%) of the intact synthetic peptide in the effluent fraction after the digestive process. Indeed, the sequence of the synthetic peptide showed four cleavage sites for pepsin and trypsin. Testing samples at 40, 60 and 120 min confirmed these results and showed that the synthetic peptide was sensitive to the digestive process under in vitro conditions. 

## 3. Discussion

In this study, AMPs with potential valorization as biopreservatives were identified in mackerel hydrolysate obtained from poorly valorized whole mackerel. This fish resource is mainly used as bait or for human consumption [16], but mackerel could also be a source of value-added products such as fish protein hydrolysates. Mackerel hydrolysate was produced by enzymatic digestion using the commercial food-grade enzyme mixture Protamex^®^, and fractionation. These steps generated peptidic compounds of molecular weight less than 10 kDa. This process was previously developed by [4] on Atlantic mackerel. As shown by Beaulieu et al., (2009), the fraction obtained was largely composed of small peptides (ranging between 7000 Da and 100 Da) rich in glutamic and aspartic acids [1]. 

This fraction was investigated for antimicrobial activity after purification. The protein hydrolysis process generated small peptides, including AMPs with strong activity against a broad spectrum of microorganisms [17]. After purification and MS/MS analyses, four peptide sequences with high homology to known AMPs were identified among the 46 peptide sequences presented in peak A, originating from 12 different precursor proteins. It is possible that the peptides were co-eluted, but then separated during the LC-MS/MS, because the ACN gradient and the column were different, which would explain the high number of peptides in peak A. AMPs from a hydrolysate of Atlantic mackerel by-products were previously identified and characterized but their sequences are different from those identified in the present study [7]. Three of the peptide sequences identified in the present study ((2), LILLILLLLKLLLLLI; (3), LLILLLLKLLLLLI and (4) LLILLLLLLILLLILLPF) were rich in leucine. Leucine-rich repeat sequences have been found in antibacterial peptides originating from macroalga hydrolysate [18] and are known to increase the antimicrobial potency of AMPs [19]. In addition, both peptide sequences (2) and (3) originated from the precursor protein N-acetylmuramoyl-l-alanine amidase. This enzyme targets bacterial peptidoglycan by hydrolyzing the amide bond linking the peptide units to the muramic acid residues of the glycan strands and participates in bacterial clearance [20]. This mode of action explains AMPs with greater activity against Gram-positive bacteria than Gram-negative bacteria. The peptide (AMGAP) (KVEIVAINDPFIDL) had a different amino acid composition than the other three peptides and possessed the highest homology with known AMPs. The AMGAP peptide was related to YFGAP, an AMP isolated from the skin of yellowfin tuna *Thunnus albacares*, and active against both Gram-positive and Gram-negative bacteria [10]. The antimicrobial fraction exhibited similar activity to YFGAP, with around tens µg/mL of peptides. Studies on the YFGAP mechanism of action revealed a bacteriostatic process rather than a lytic mechanism with pore formation, which explained the high concentrations of peptides needed to produce the antibacterial activity [10]. In addition, the GAPDH-related antimicrobial peptide was characterized from skipjack tuna, another scombrid [21]. Interestingly, it was recently reported that a YFGAP homologue was identified in a snow crab hydrolysate [22]. Thus, the peptide sequence KVEIVAINDPFIDL was chosen for synthesis due to (i) having the highest homology to an antimicrobial-peptide, (ii) its homology with an antimicrobial peptide identified from a closely related species belonging to the *Scombridae* family, and (iii) its structural stability based on in vivo half-life of 16 h or more [23].

The antibacterial activity was first confirmed using synthetic AMGAP. Then, the antibacterial activity and stability during transit through an artificial GI tract were evaluated. The MIC was determined against both human commensal flora and bacterial pathogens. The MIC values revealed that synthetic AMGAP peptide was less active than the purified RP-HPLC fraction, peak A. This difference could be explained by the synergistic action of other antimicrobial compounds present in peak A, as suggested by MS/MS results [24]. Nevertheless, *Listeria* strains were the most sensitive bacteria to the synthetic AMGAP peptide as suggested by results obtained with the C18-SPE purified fraction. The genus *Listeria* has been responsible for severe foodborne diseases in humans, especially *L. monocytogenes* [25,26]. *Listeria* occurs often in food and food processing environments [27], and the synthetic peptide identified could be used as a food-preservative to prevent bacterial development. In the food industry, other pathogens are known to contaminate food, such as *E. faecalis, E. coli, S. aureus,* and *V. parahaemolyticus* [28], which shows little sensitivity to the synthetic AMGAP peptide. To improve its antimicrobial activity, the synthetic AMGAP peptide could be modified by adding co-factor or replacing particular amino acids, as demonstrated for negatively charged peptides [29,30]. As already shown for marine anionic antibacterial peptides, the antibacterial activity increased at alkaline pH [31], and so AMGAP activity might be improved at higher pH. Conversely, the synthetic peptide also exhibited activity against common human organisms such as *M. luteus*, *L. acidophilus* and *B. thetaiotaomicron*. Nonetheless, the MICs were half the *Listeria* MIC values, meaning that at equivalent concentrations, the synthetic peptide could inhibit *Listeria* growth without affecting *M. luteus*, *L. acidophilus* and *B. thetaiotaomicron*. Lactobacillus, which is an important component of the human microbiota, produced antimicrobial substances active against a wide range of bacteria, such as *Listeria* [32]. Thus, it is important to limit the action of AMPs on normal flora [33]. 

Food additives such as AMPs must be safe for the human microbiota and the environment. Consequently, knowing the digestibility of AMPs in the human gastrointestinal tract is critical and should not be underestimated [34]. The TIM-1 model used to evaluate the stability of the synthetic peptide simulated the major GI stress conditions, such as gastric acid environment, excretion of proteolytic enzymes and bile salts, and peristaltic movements [35]. Using TIM-1, we have shown that the synthetic AMGAP peptide would not be an environmental concern because it was rapidly digested (after 20 min) in the first stage of the digestive process (stomach). This rapid digestibility was attributable to the presence of both pepsin and trypsin in the stomach compartment, which cleaved *Lys*^1^*Val*, *Pro*^10^*Phe*, *Asp*^13^*Leu* and *Leu*^14^ in the synthetic peptide. A similar result was observed with the pediocin PA-1, where gastric pepsin, combined with the low pH, reduced its stability [36]. Therefore, the digestibility of the synthetic peptide in the stomach compartment and its low activity against bacteria from the human flora, suggest that: (i) human commensal flora will not be impacted, and (ii) creation of antibacterial resistance will be very limited. 

## 4. Materials and Methods 

### 4.1. Mackerel Hydrolysate

#### 4.1.1. Hydrolysate Process

Mackerel hydrolysate fractions were produced at Merinov (Gaspé, QC, Canada) according to a procedure adapted from [4]. Briefly, 100 kg of ground mackerel fished in 2016 was added to an equal amount of demineralized water (*w*/*w*), and the total volume was heated to 40–43 °C. Then, 100 g Protamex (Novozymes, Bagsvaerd, Denmark) was added to start the hydrolysis. After 150 min of hydrolysis at 40–43 °C, the tank temperature increased to 90 °C, to inactivate proteases. The liquid fraction was decanted using a clarifying decanter and then centrifuged at 11,000 g to separate suspended insoluble matter and lipids from the hydrolysate. The hydrolysate was then ultrafiltered (spiral membrane with molecular weight cut off of 10 kDa) to separate proteins and peptides according to molecular mass. Permeate from the 10 kDa membrane was nano-filtered at 200 Da (Model R, GEA filtration, Hudson, WI, USA) to obtain a 10 kDa–200 Da retentate. The nano-filtration retentate was spray-dried and stored at 4 °C until analyzed.

#### 4.1.2. Chemical Composition

The chemical composition of mackerel hydrolysates was determined by measuring moisture, minerals (ash), lipids and protein content. Moisture and minerals were measured using the official methods of analysis of the Association of Official Analytical Chemists [37]. Lipids were analyzed using the modified Bligh and Dyer method [38]. Proteins were determined by the Kjeldahl method (nitrogen × 6.25) adapted from the A.O.A.C official method [37]. The dry mass of the starting material and all fractions were determined by subtracting the moisture content from the wet mass.

#### 4.1.3. Amino Acid Composition

The amino acid composition of mackerel hydrolysate was determined as described by [4]. Analyses were based on the determination of amino acids resistant to acid hydrolysis using the AccQ-Tag amino acid analysis procedure (Waters). Briefly, amino acids were separated by RP-HPLC and quantified by fluorescence detection.

#### 4.1.4. Molecular Weight Distribution of the Hydrolysate

Molecular weight distribution was performed on the hydrolysate sample by gel permeation chromatography on a Superdex™ Peptide 10/300 GL column (GE Healthcare, Baie-D’Urfé, Qc, Canada) using a FPLC system (Akta Avant, GE Healthcare, Baie-D’Urfé, Qc, Canada). The column was calibrated according to the manufacturer’s guidelines with proteins of known molecular weight (reference samples). The mobile phase consisted of 50 mM sodium phosphate buffer containing 150 mM of NaCl at pH 7.0. A mixture of glycine (1.00 mg/mL), aprotinin (0.200 mg/mL), ribonuclease A (0.200 mg/mL), and bovine serum albumin (0.650 mg/mL) (Sigma, Oakville, ON, Canada) was injected (0.500 mL) on the column for the calibration. This yielded a near linear correlation between the retention time and the log of the molecular mass of proteins in the range of 6512 Da to 75 Da. Briefly, the hydrolysate was solubilized at 1 mg/mL with the mobile phase and then injected (0.500 mL) on the column. Samples were eluted (isocratic) at a flow rate of 0.8 mL/min. Proteins/peptides were detected by monitoring the absorbance at 214 nm.

### 4.2. Identification of Antibacterial Peptide from the Mackerel Hydrolysate

#### 4.2.1. Microbial Strains and Culture Conditions

The indicator strains used in this study, their origins and the culture media are listed in Table 5. All strains were maintained in 50% glycerol at −80 °C until use. 

#### 4.2.2. Antibacterial Activity 

Antibacterial activity was screened using the well diffusion assay. Bacteria were routinely cultured under optimal growth conditions (temperature and broth). For the assay, the bacterial inoculum was freshly prepared in new broth at a concentration of approximately 10^6^ CFU/mL, based on the optical density at 600 nm. Samples (20 µL) were loaded into 4 mm wells in specific agar medium inoculated with the bacterial target at 5 × 10^5^ CFU/plate. Results were recorded as the diameter of the inhibition zone after 24 h at the specific growth temperature of the indicator strain.

The antibacterial activities of both whole mackerel hydrolysate and SPE fractions were quantified using a previously described microtitration method [39]. Briefly, twofold serial dilutions of samples (100 µL) were made in microplate wells (96-well Microtest, Becton Dickinson Lab-ware, Sparks, MD, USA) containing 100 µL of the appropriate culture medium. From an overnight culture grown under the specific growth conditions of the target strains (Table 1), each well was fed (50 µL) with the strains diluted in the appropriate culture medium to 1% (10^6^ CFU/mL). This suspension brought the final volume in each well to 150 µL. The microplates were incubated at the appropriate bacterial growth temperature and the absorbance at 600 nm was recorded for 20 h using an Infinite F200 PRO photometer (TECAN US Inc., Durham, NC, USA). Activity was expressed in arbitrary units per milliliter (AU/mL) and calculated as AU/mL = 2^n^ × (1000/150), with *n* = number of inhibited wells [40]. 

The MIC of the antibacterial peptides was determined against a panel of bacterial targets (Table 1). This assay was conducted in sterile 96-well microtiter plates (Nunc-167314) as described by [41]. Briefly, a two-fold dilution series of the compound stock solution (1.050 mM, 50 µL) was prepared in Mueller-Hinton Broth or Brucella Broth (with vitamin K1, 1 µg/mL and lyzed horse blood, 5%) in which the bacterial target (50 µL) was inoculated (5.10^5^ CFU/mL, final concentration). The MIC was defined as the lowest antibacterial compound concentration that inhibited visible growth of the target bacterium after incubation for 24 or 48h at the appropriate growth temperature. 

### 4.3. Purification

#### 4.3.1. Solid Phase Extraction

A SPE-C18 (2G cartridges, Waters, Canada) was applied to the solubilized hydrolysate (0.25 g/mL in ultrapure water) as a preliminary concentration step. Briefly, after equilibration in 0.07% trifluoroacetic acid (TFA), hydrolysate (50 mL) was loaded and sequentially eluted using ACN 0.1% TFA at 10%, 55% and 70% ACN. Dried fractions were reconstituted with 0.1% TFA in 50% ACN and assayed for antibacterial activity as described below. 

#### 4.3.2. Liquid Chromatography

Bioactive fractions were subjected to a two-step RP-HPLC procedure conducted on an Agilent 1100 Series HPLC Value system (Agilent Technologies, Hewlett-Packard-Strasse 8, Germany) using a Peptide XB-C18 10 (5 µm, 250 × 4.6 mm, Aeris™, Canada). For these two steps of purification, the mobile phase consisted of buffer A (H_2_O, 5mM HCl) and buffer B (ACN). The column was heated at 40 °C and elution was performed using a linear gradient of buffer B at 0.8 mL/min. The gradient program started with 70% of buffer B for 3 min before increasing to 90% over 20 min. Solvent was maintained at 90% of buffer B for 1 min before returning to starting conditions over 2 min. The injection volume was 100 µL and the elution was monitored by UV absorbance at 214 nm. Every 5 min, fractions were collected and then dried. Before being tested for their antibacterial activity, the fractions were dissolved in water with acetonitrile (50:50, *v*/*v*).

### 4.4. Structural Identification of Antibacterial Peptide 

#### 4.4.1. LC-MS/MS Analysis

Mass spectrometry analyses were performed by the Proteomics Platform of the CHU de Québec Research Center (Québec, Qc, Canada). Peptide samples were desalted on a C18 Empore filter (Stage-Tip) then analyzed by LC-MS/MS. The experiments were performed with Ekspert NanoLC425 (Eksigent) coupled to a 5600+ mass spectrometer (Sciex, Framingham, MA, USA) equipped with a nanoelectrospray ion source. Peptides (approximately 1 ug) were separated on picofrit columns (Reprosil 3u, 120A C18, 15 cm × 0.075 mm internal diameter). Peptides were eluted with a linear gradient from 5% to 90% of solvent B (acetonitrile, 0.1% formic acid) in 90 min, at 300 nL/min. Mass spectra were acquired using a data-dependent acquisition mode using Analyst software version 1.7. Each full scan mass spectrum (400 to 1250 *m*/*z*) was followed by collision-induced dissociation of the twenty most intense ions. Dynamic exclusion was set for a period of 12 sec and a tolerance of 50 mDa.

#### 4.4.2. Database Searching

MGF peak list files were created using Protein Pilot version 5.0 software (Sciex, Concord, ON, Canada). These MGF sample files were then analyzed using Mascot (Matrix Science, London, UK; Version 2.5.1). Mascot searched the TAX_ Percomorphaceae_CI_1489872_20170406 database (86,210 entries) assuming the digestion non-specific enzyme. Mascot was searched with a fragment ion mass tolerance of 0.100 Da and a parent ion tolerance of 0.100 Da. Glu->pyro-Glu of the N-terminus, gln->pyro-Glu of the N-terminus, deamidated of asparagine and glutamine and oxidation of methionine were specified in Mascot as variable modifications.

#### 4.4.3. Criteria for Protein Identification

Scaffold (Version Scaffold_4.7.3, Proteome Software Inc., Portland, OR, USA) was used to validate MS/MS-based peptide and protein identification. Peptide identification was accepted if it could be established at greater than 95.0% probability by the Peptide Prophet algorithm [42] with Scaffold delta-mass correction. Protein identification was accepted if it could be established at greater than 95.0% probability and contained at least 2 identified peptides. Protein probabilities were assigned by the Protein Prophet algorithm [43]. Proteins that contained similar peptides and could not be differentiated based on MS/MS analysis alone were grouped to satisfy the principles of parsimony. 

### 4.5. Digestibility of Antibacterial Peptide

#### 4.5.1. Digestion and Sampling

The dynamic GI TIM-1 Model (TNO Nutrition and Food Research Institute, Zeist, the Netherlands) described previously by [44] was used for evaluation of peptide digestibility. Gastric pH began at 4.5 and decreased gradually to 3.6 after 20 min, 2.7 after 40 min and 2.0 after 60 min of digestion by injecting 0.5 mol/L HCl, finally reaching 1.7 after 120 min. The pH of the duodenal, jejunal and ileal compartments was adjusted to 6, 6.8 and 7.2, respectively, by injecting 0.5 mol/L sodium bicarbonate solution. Gastric secretions consisted of pepsin (0.163 mg/mL) from porcine gastric mucosa (EC 3.4.23.1; Sigma-Aldrich Canada Ltd., Oakville, ON, Canada) and lipase (0.133 mg/mL) from Rhizopus oryzae (EC 3.1.1.3; Amano Pharmaceuticals, Nagoya, Japan), both in an electrolyte solution (NaCl, 6.2 g/L; KCl, 2.2 g/L; CaCl_2_, 0.3 g/L; NaHCO_3_, 1.5 g/L) delivered at a flow rate of 0.25 mL/min. Duodenal secretions consisted of 4% pancreatin solution (8xUSP, Pancrex V powder; Paines and Byrne, Greenford, UK) and 4% porcine bile extract (Sigma-Aldrich Canada Ltd) as well as the small intestine electrolyte solution (NaCl, 5.0 g/L; KCl, 0.6 g/L; CaCl_2_, 0.3 g/L; pH 7.0) were injected, at 0.25, 0.5 and 0.25 mL/min, respectively, and the total injected volumes were logged. Before starting the digestion processing, 1 mL of trypsin solution (2 mg/mL) was added to the duodenal compartment, then 30 mg of synthetic peptide (Biobasic, Markham, ON, Canada) was dissolved into 300 mL of pure water and injected into the stomach compartment. Samples were withdrawn from stomach, duodenum and ileal-delivered effluent after 0, 20, 40, 60 and 120 min of digestion.

#### 4.5.2. Analysis of the GAPDH-Related Derived Peptide Degradation

To check the digestibility of the synthetic GAPDH-related derived peptide, TIM samples were separated by SPE-C18 before analysis by FPLC with ionic exchange column (IEX). Each sample (10 mL) was loaded on a SEP cartridge and eluted with 10% and 90% ACN (*v*/*v*) (in demineralized H_2_O). After evaporation of ACN with a Speed-Vac concentrator (Thermo Savant Instruments Inc., Waltham, MA, USA), SPE fractions were solubilized in 1 mL of buffer A (Tris, 50 mM, pH 8). Detection of the synthetic peptide was conducted on a HiTrap Q FF column (1 mL, GE, Mississauga, ON, Canada), where 500 µL of each fraction was injected. The column was eluted with a linear gradient of buffer B (buffer A containing 1 M NaCl) from 0 to 50% in a 20-column volume. The absorbance was recorded at 214 nm and collected fractions were screened for sequence peptides by LC-MS/MS analysis as described above. 

## 5. Conclusions

In this study, we investigated mackerel hydrolysate for its potential as a bio-preservative. Screening and characterization of the hydrolysate for antibacterial peptides identified four peptide sequences. These four sequences showed high homology with known AMPs. Based on these results, synthesis of the most promising peptide allowed us to validate the first steps for potential valorization as a bio-preservative. By evaluating antibacterial activity against important bacteria from both the food industry and human flora, this synthetic GAPDH-related derived peptide (AMGAP) was found to inhibit the growth of food spoilage organisms without impacting normal human flora. It remains to elucidate the AMGAP mode of action to ensure its effectiveness against specific pathogens. As a bio-preservative, this peptide must be safe for human consumption and for the environment. Human digestion simulated by TIM-1 degraded the synthetic AMGAP peptide in the stomach compartment. Based on these results, this novel AMGAP peptide, specifically purified or directly synthesized, would be a good candidate for valorization as a bio-preservative but further investigation is needed to ensure its global safety. Therefore, mackerel hydrolysate could be used as a high value product having bio-preservative properties. More generally, this study revealed that Atlantic mackerel hydrolysate is a promising source of AMPs that can be used for new applications in food safety.

## Figures and Tables

**Table 1:** Total amino acids in the hydrolysate (g/100 g) from processing of Atlantic mackerel (10,000–200 Da retentate). Analyses were conducted in duplicate.

Amino Acids	Mackerel Hydrolysate
Glutamic acid	13.23 ± 0.70
Aspartic acid	8.31 ± 0.28
Lysine	7.14 ± 0.46
Leucine	5.37 ± 0.13
Glycine	5.06 ± 0.29
Arginine	4.71 ± 0.15
Alanine	4.97 ± 0.07
Proline	3.43 ± 0.19
Valine	3.39 ± 0.25
Threonine	3.40 ± 0.09
Serine	3.27 ± 0.20
Histidine	3.17 ± 0.26
Phenylalanine	2.30 ± 0.19
Isoleucine	2.51 ± 0.21
Tyrosine	2.04 ± 0.18
Methionine	1.88 ± 0.16
Taurine	0.48 ± 0.03
Cysteine	0.27 ± 0.03
Tryptophan	0.64 ± 0.02
**Total**	75.57

**Table 2:** Purification of antibacterial peptides from hydrolysate of whole mackerel.

Purification Step	Volume (mL)	Total Protein (mg)	Total Activity (AU)	Specific Activity (AU/mg)	Increase in Specific Activity (fold)	Activity Recovery (%)
**Solubilized hydrolysate**	50	1.25 × 10^3^	1.00 × 10^6^	8.00 × 10^1^	/	100
**SPE-C18 eluate**	1.0	1.48 × 10^0^	6.40 × 10^5^	4.32 × 10^5^	5405	64
**First RP-HPLC eluate**	0.5	1.70 × 10^−2^	2.00 × 10^4^	1.18 × 10^6^	14,706	2
**Second RP-HPLC eluate**	0.3	4.00 × 10^−3^	6.00 × 10^3^	1.67 × 10^6^	20,833	1

**Table 3:** Peptide fragments identified by MS/MS analysis, with more than 75% homology to antibacterial proteins from CAMP. Peptide sequence properties and their related precursor proteins were obtained from ExPASY and Scaffold (protein-threshold probability > 95%) analyses, respectively.

Scaffold Analysis			ExPASy Analysis				CAMP Database			
	Peptide Sequences	Precursor Proteins	Charge	GRAVY Index	Stability	pI	Antibacterial-Peptide Related	Access Number	Homology	Organisms
(1)	KVEIVAINDPFIDL	Glyceraldehyde-3-phosphate dehydrogenase	−2	0.771	Stable	4.03	YFGAP	CAMPSQ3690	89%	*Thunnus albacares*
(2)	LILLILLLLKLLLLLI	N-acetylmuramoyl-L-alanine amidase	+1	3.450	Stable	8.75	Beta-defensin	CAMPSQ4887	75%	*Saguinus oedipus*
(3)	LLILLLLKLLLLLI		+1	3.350	Stable	8.75		CAMPSQ4886	75%	*Callithrix jacchus*
(4)	LLILLLLLLILLLILLPF	Syndecan domain	0	3.561	Unstable	5.52	Beta-defensin	CAMPSQ4886	75%	*Capra hircus*

**Table 4:** MIC of the synthetic peptide (AMGAP) expressed in mM. ND, non-determined.

*Genus*	*Species*	Strains	MIC (mM)
*Bacillus*	*megaterium*	B301	>1.050
*Blautia*	*coccoides*	ATCC 29236	>1.050
*Bifidobacterium*	*infantis*	ATCC 15697	>1.050
*Enterococcus*	*faecalis*	ATCC 29212	>1.050
*Enterococcus*	*faecium*	L1	>1.050
*Lactobacillus*	*acidophilus*	ATCC 4356	0.263
*Lactobacillus*	*salivarius*	16	ND
*Listeria*	*ivanovii*	ATCC 19119	0.131
*Listeria*	*monocytogenes*	ATCC 15313	0.131
*Micrococcus*	*luteus*	272	0.263
*Staphylococcus*	*aureus*	ATCC 25923	>1.050
*Bacteroides*	*thetaiotaomicron*	ATCC 29741	0.263
*Escherichia*	*coli*	ATCC 25922	>1.050
*Pseudomonas*	*aeruginosa*	ATCC 27853	>1.050
*Vibrio*	*parahaemolyticus*	ALN-0312	>1.050

**Table 5:** Bacterial targets used for screening of antibacterial activity from hydrolysate. BHI, Brain Heart Infusion; LB, Luria-Bertani; TSB, Tryptone Soy Broth; YE, Yeast Extract; YEG, Yeast Extract Glucose.

Target strains					Growth Conditions
Genus	*Species*	Collection	Type	Ecological Function	Media
*Bacillus* ^†^	*megaterium*	B301	Gram ^†^	Non-pathogen	TSB
*Blautia*	*coccoides*	ATCC 29236		Human Flora	BHI + Cysteine (0.05%)
*Bifidobacterium*	*infantis*	ATCC 15697		Human probiotic	BHI + Cysteine (0.05%)
*Enterococcus*	*faecalis*	ATCC 27212		Human pathogen	BHI
*Enterococcus* ^†^	*faecium*	L1		Human pathogen	BHI
*Lactobacillus*	*acidophilus*	ATCC 4356		Human probiotic	TSB+YE (0.6%)
*Lactobacillus* ^†^	*salivarius*	16		Human probiotic	BHI + Cysteine (0.05%)
*Listeria*	*ivanovii*	ATCC 19119		Human pathogen	TSB+YE (0.6%)
*Listeria*	*monocytogenes*	ATCC 15313		Human pathogen	MRS
*Micrococcus* ^†^	*luteus*	272		Non-pathogen	TSB
*Staphylococcus*	*aureus*	ATCC 25923		Human pathogen	BHI
*Bacteroides*	*thetaiotaomicron*	ATCC 29741		Human pathogen	BHI + Cysteine (0.05%)
*Escherichia*	*coli*	ATCC 25922		Human pathogen	LB
*Pseudomonas*	*aeruginosa*	ATCC 27853		Human pathogen	TSB
*Vibrio*	*parahaemolyticus*	ALN-0312		Human pathogen	BHI + Sea salt (3%)

^†^ Laboratoire de Microbiologie Alimentaire (LMA) strain collection (Laval University, QC, Canada).
